# Remarks on the Pretended Recent Improvements in the Practice of Medicine, Especially in Fever

**Published:** 1819-08

**Authors:** 


FOR THE LONDON MEDICAL AND PHYSICAL JOURNAL.

### *Remarks on the pretended recent Improvements in the Practice of Medicine, especially in Fever.* By Senex.

THROUGH the medium of your useful Journal, and two or three other periodical publications, I am enabled to see what is going on in the medical world; and can, by means of your extracts, &c. make myself tolerably well acquainted with all the modern improvements in the profession, to which I have been for nearly forty-five years daily devoted.

You must, sir, I think, be well aware how mortifying it must be to an old man like me, to see my highly respected masters, former acquaintances, and favourite remedies, treated with such contempt as all the modern writers on fever have indulged themselves in; and one would really think, that none of us, who practised forty years ago, could possibly have had common sense or discrimination in the management of fever, when we read of the superior methods which these youngsters fancy they have discovered in treating the several epidemic diseases of the day. Not content with merely introducing their treatment of fever, by prescribing venesection, calomel, and purges, as totally new, they seem to think that we had not the judgment to perceive the nature of the disease, but that we must have been totally devoid of all discernment whilst giving bark in fevers. But, sir, I think I may venture to say, that not one of the modern writers on epidemics was probably in existence then, certainly not as physicians: how, then, does he know what were the existing circumstances of climate, diet, clothing, &c. of that day. Might not the epidemics of former times have been variously modified according to these circumstances; and was it not the business of a practitioner to adapt his remedies to the times, according to the excellent advice and practice of Sydenham, always to feel his way when an epidemic appeared? I have, for forty years and upwards, done so. I have no theoretical system nor specific recipes for diseases; nor do I like to be asked for their names. Though long an attentive pupil of both the Hunters, of Fordyce, and Saunders, and have sat many an hour under Cullen and Monro, attended patiently Harry Home and John Brown, read Morgagni, Haller, &c.; still, sir, I think myself justified in attributing my good fortune to the daily uninterrupted use of my own reason, through a period of great energy and activity, unimpaired, in the opinion of a large circle of friends, at this hour. I have lived long enough to see a great variety of epidemic fevers, with many changes of name. In the winters of the years 75 and 6, I attended hundreds of poor in continued fever, then epidemic and contagious, under the then prevalent appellation of low nervous fever: these were generally treated, and successfully treated, by nothing but tartar emetic and chalk, with an occasional dose of jalap; taking the hint (for "*fas est et ab hoste doceri*,") from the then just-introduced Dr. James's powder. In 78-9, I had as many in ague, the then general epidemic: these were all cured by bark, preceded by an emetic.

I have always treated puerperal diseases according to my observation of symptoms; have bled for five-and-twenty years, and purged for upwards of thirty, as freely as any modern: but I neither bleed nor purge all whom I am called upon to attend. The free use of calomel in bilious complaints, I learned from that most excellent practitioner, the late Dr. Clark, of Newcastle, nearly thirty years ago, long before some of those who boast of having introduced it into practice were in the profession. Contempt for the knowledge of predecessors, or older times, is not peculiar to the young practitioner in medicine. I have been treated with very apparent derision by men who were born within the last twenty-five years, for asserting that I have eaten as fine grapes and peaches, grown in the open air in this country, as are now produced in the most improved hot-houses. May not, then, the seasons and other circumstances have produced changes in the diseases as well as fruits; and, as it is now improbable that the rains of summer should be so abundant as to fill our ditches deep enough to prevent the decomposition of water, may we not expect, this autumn, again to have ague an epidemic, and may we not then find it again necessary to have recourse to bark.

Marsh miasma, the long-acknowledged source of remittent and intermittent fever, is not the result of simple exhaling moisture, (for then the sailor would be the victim,) but arises from decomposition, when the sun acts upon the mud and refuse of decaying vegetable matter in moist places. Almost all our summers and winters, for a great many years, have had such surfaces always covered deep with water, and then simple evaporation takes place only; but the last dry summer, and this wonderful winter, in which almost all our springs have failed, having left all such places uncovered, except when showers have just fallen, may we not expect that such a mal aria will be produced as may render ague again epidemic. Then, our modern typhous writers will have to learn a new mode of treatment, and in good time discover, that successful practice, through a long life, does not depend on any fine-drawn distinctions of one or two epidemics; but must be the result of patient and diligent observation of circumstances, adapting means to ends, as experience shall direct.

*Yorkshire; June* 1, 1819.

